# Wnt Pathway Activation in Long Term Remnant Rat Model

**DOI:** 10.1155/2014/324713

**Published:** 2014-06-05

**Authors:** E. Banon-Maneus, J. Rovira, M. J. Ramirez-Bajo, D. Moya-Rull, N. Hierro-Garcia, S. Takenaka, F. Diekmann, O. Eickelberg, M. Königshoff, J. M. Campistol

**Affiliations:** ^1^Comprehensive Pneumology Center, University Hospital Großhadern, Ludwig-Maximilians-University, 81337 Munich, Germany; ^2^Fundació Clínic, Laboratori Experimental de Nefrologia i Transplantament (LENIT), CELLEX 2B, C/Casanova 143, 08036 Barcelona, Spain; ^3^IDIBAPS, Laboratori Experimental de Nefrologia i Transplantament (LENIT), CELLEX 2B, 08036 Barcelona, Spain; ^4^Hospital Clínic, Department of Nephrology and Kidney Transplantation, 08036 Barcelona, Spain

## Abstract

Progression of chronic kidney disease (CKD) is characterized by deposition of extracellular matrix. This is an irreversible process that leads to tubulointerstitial fibrosis and finally loss of kidney function. Wnt/**β**-catenin pathway was reported to be aberrantly activated in the progressive damage associated with chronic organ failure. Extensive renal ablation is an experimental model widely used to gain insight into the mechanisms responsible for the development of CKD, but it was not evaluated for Wnt/**β**-catenin pathway. This study aimed to elucidate if the rat 5/6 renal mass reduction model (RMR) is a good model for the Wnt/**β**-catenin activation and possible next modulation. RMR model was evaluated at 12 and 18 weeks after the surgery, when CKD is close to end-stage kidney disease demonstrated by molecular and histological studies. Wnt pathway components were analyzed at mRNA and protein level. Our results demonstrate that Wnt pathway is active by increase of **β**-catenin at mRNA level and nuclear translocation in tubular epithelium as well as some target genes. These results validate the RMR model for future modulation of Wnt pathway, starting at shorter time after the surgery.

## 1. Introduction

Progression of chronic kidney disease (CKD) is characterized by deposition of extracellular matrix (ECM). This is an irreversible process that leads to tubulointerstitial fibrosis and finally loss of kidney function. It is widely accepted that epithelial-to-mesenchymal transition (EMT) is the main factor responsible for organ fibrosis. EMT is defined as phenotypic change of fully differentiated epithelial cells to matrix-producing fibroblasts [1].

Several studies have suggested that specific signal pathways may affect the development of fibrosis in the kidney and contribute to epithelial cell injury and fibroplasia. Some of the signaling mediators involved include proinflammatory and profibrotic cytokines produced by infiltrating and resident cells, such as interferon-*γ*, tumor necrosis factor-*α*, interleukin-1-*β*, and transforming growth factor-*β*-1 (TGF-*β*) [2].

The TGF-*β* signaling pathway plays a pivotal role in embryonic development and organogenesis and is a key fibrogenic cytokine in several fibrotic diseases of the kidney, lung, and other organs. Campistol et al. have previously demonstrated that TGF-*β* serum levels were significantly higher in kidney transplant patients with interstitial fibrosis and tubular atrophy than in those with normal renal function [3, 4].

TGF-*β* has been shown to be critical for extracellular matrix turnover and for cellular plasticity, such as epithelial-to-mesenchymal transition. This transition represents the phenotypic, reversible switching of epithelial to fibroblast-like cells and may play a major role in the pathogenesis of CKD, increasing the number of activated fibroblasts derived from epithelial cells [5–7]. The role of TGF-*β* in fibrogenic processes and renal fibrosis has been widely described, as has been the role of crosstalk between TGF-*β* and Wnt/*β*-catenin pathways in cell fate determination during embryonic development and in the adult [8]. Crosstalk between two pathways could be carried out by transregulation, whereby one pathway regulates specific components of the other or through cross-communication between components of the pathways. For example, the stability of the intracellular signal transducers of the TGF-*β* pathway, the SMAD proteins, can also be regulated by axin, a regulator of Wnt signaling. However, there is little evidence of interdependence or retroregulation between the components of the two pathways in transcriptional regulation of target genes in mammals, particularly in the context of epithelial-to-mesenchymal transition and organ fibrosis [8].

Over the last years, the Wnt/*β*-catenin pathway was reported to be aberrantly activated in the progressive damage associated with chronic organ failure [9, 10]. Wnts are a family of 19 proteins essential for organ development, a process that has been described to be recapitulated in organ failure. Canonical Wnt pathway involves the cytoplasmic protein *β*-catenin. In the absence of Frizzled (FZD) receptor binding of Wnt ligands, *β*-catenin is constitutively phosphorylated by glycogen sintase kinase-3*β* (GSK-3*β*) and subsequently degrades by the complex composed of the adenomatous polyposis coli protein (APC) and axin. In the presence of Wnt ligand binding to a FZD receptor and a low density receptor (LRP) coreceptor leads to the phosphorylation of disheveled protein in the cytoplasm (DVL). The activated DVL binds to axin in order to antagonize the action of GSK-3*β*; subsequently *β*-catenin phosphorylation and ubiquitination are inhibited. This causes a citoplasmatic accumulation of *β*-catenin in the cytoplasm and translocation to the nucleus where it regulates target gene expression through interaction with members of the TCF/LEF family of HGM-domain transcription factors [9, 10].

Increased Wnt/*β*-catenin signaling has been shown to be involved in epithelial cell injury and hyperplasia, as well as in impaired epithelial-mesenchymal crosstalk in fibrosis. Modulation of Wnt signaling led to an attenuation of lung fibrosis* in vivo* [9], while altered expression of Wnt signaling components has been reported in several renal diseases [11] and animal models, such as rat chronic allograft dysfunction model [2], unilateral ureteral obstruction model [12], lupus nephritis [13], or diabetic nephropathy [14].

Several CKD animal models have been used to study the mechanisms responsible for the disease progression. Extensive renal ablation is an experimental model widely used to gain insight into the mechanisms responsible for the development of CKD since it causes a combination of glomerular hemodynamic alterations and nonhemodynamic events that result in progressive glomerulosclerosis, tubulointerstitial damage, proteinuria, and renal functional impairment in the remnant kidney. There is early glomerulosclerosis by week 4, with segmental sclerosis with tubulointerstitial fibrosis by week 8 [15, 16].

This study aimed to elucidate if the 5/6 renal mass reduction model is a good model for the Wnt/*β*-catenin activation and possible next modulation. To do this, we used 5/6 renal mass reduction model animals and we sacrificed animals at 12 and 18 weeks after the surgery, when CKD is well established, and then we characterized the fibrosis establishment and the Wnt activation.

## 2. Methods

### 2.1. Antibodies and Reagents

In this study, the following antibodies were used: total *β*-catenin (number 9562), phospho-S9- and total GSK-3*β* (numbers 9336 and 9315), phospho- and total LRP6 (numbers 2568 and 2560), and phospho-SMAD3 (C25A9) (all from Cell Signaling Technology, Beverly, MA); Wnt1 (600-401-A37) (Rockland, Gilbertsville, PA); *α*-SMA (A2547); Ca^2+^ATPase (A7952); cyclin D1 (C7464); GADPH (G9545); *β*-actin (A3854); Pan CK (F3418); Desmin (D1033) and SIS3 (S0447) (all from Sigma-Aldrich, Saint Louis, MO); FBPase (sc-166097) and factor VIII (sc-27647) (from Santa Cruz Biotechnology, Santa Cruz, CA); Alexa Fluor 568 phalloidin (A-12380) (Invitrogen); vimentin (AB1620) and TGF-*β* (MAB1032) (from Millipore, Billerica, MA).

### 2.2. Animal Model: The 5/6 Renal Mass Reduction

#### 2.2.1. Animals

Male Wistar rats (Charles River Laboratories España, Barcelona, Spain) weighing approximately 225 g were used. The rats were kept at a constant temperature and humidity and at a 12 h light/dark cycle. The animals had free access to standard rat chow (Harlan Interfauna Iberica, S.L., Barcelona, Spain) and water.

This study was approved by and conducted according to the guidelines of the Local Animal Ethics Committee (Comitè Ètic d'Experimentació Animal, CEEA, Decret 214/97, Catalunya, Spain).

#### 2.2.2. Experimental Design

Animals (*n* = 33) were randomly assigned to two groups according to the surgical intervention. One group was assigned to undergo renal mass reduction (nephrectomized group (Nx)) and the other to have a sham operation (sham group (S)). Surgical procedures were performed under general anesthesia with isoflurane (Forane; Abbott Laboratories, S.A., Madrid, Spain). After an abdominal incision, the left kidney was exposed and separated from the adrenal gland. The lower and upper poles of the left kidney were frozen by application of a cylinder of dry ice of standard size for 2 minutes on each pole. After one week, the right kidney was removed. The sham group rats underwent the same abdominal incision and manipulation of the left and the right kidneys without tissue destruction as described by Rovira et al. [15, 16].

### 2.3. Renal Function Parameters

Serum creatinine, BUN, proteinuria, urinary creatinine, and proteinuria/creatinine ratios were determined 1-2 days before sacrifice. To collect urine samples the rats were housed in metabolic cages separately for 24 h.

### 2.4. Tissue Collection

At the end of the study, the rats were killed and kidney samples were harvested. Central slide from kidney was fixed in formalin and embedded in paraffin by routine methods. The remaining tissue was snap-frozen and stored at −80° until use.

### 2.5. Reverse Transcription and Quantitative Real-Time PCR

Total RNA was extracted by using a Perfect Pure RNA fibrous Tissue Kit (5PRIME, Hamburg, Germany) from renal tissue (human and rat) and by using RNeasy Mini Kit (Qiagen, Hilden, Germany) extraction kits according to the manufacturer's protocol. cDNAs were generated by reverse transcription using MuLV reverse transcriptase (Roche, Indianapolis). Quantitative (q)RT-PCR was performed using Light Cycler 480 SyBr Green I master (Roche), as previously described. r_RPL19, ubiquitously and equally expressed gene free of pseudogenes, was used as a reference gene in all qRT-PCR reactions. PCR was performed using the primers at a final concentration of 200 nM. The relative transcript abundance of a gene is expressed in DCt values (DCt = Ct_reference_ − Ct_target_). Relative changes in transcript levels compared with controls are expressed in DDCt values (DDCt = DCt_treated_ − DCt_control_). All DDCt values correspond approximately to the binary logarithm of the fold change, as mentioned in Section 3.

### 2.6. Protein Extraction from Tissue

Renal rat tissue was homogenized in extraction buffer QProteome Mammalian Protein Preparation Kit (QIAGEN), and the whole proteins were extracted by centrifugation (14,000 rpm) for 10 min at 4°C, as recommended by the supplier. Proteins were quantified using the Micro BCA Protein Assay Kit (PIERCE).

### 2.7. Western Blot

Samples containing 30 *μ*g of protein were separated by electrophoresis on SDS polyacrylamide gels. The separated proteins were transferred to PVDF membranes (BioRad), blocked with 5% skimmed milk or BSA depending on customer indication, and incubated with the indicated antibodies. The proteins were then visualized by enhanced chemiluminescence detection (ECL, Pierce) with Image Quant LASS 4000 (GE Healthcare). Densitometry quantification was done using Image J.

### 2.8. Histology

Human biopsies and rat kidneys were placed in 4% (w/v) paraformaldehyde after explantation and were processed for paraffin embedding. Sections (3 *μ*m) were cut and mounted on slides. Light microscopic evaluation was performed on sections stained by Masson trichrome to evaluate fibrotic changes in representative renal allografts [3].

### 2.9. Immunohistochemistry

The 3 *μ*m sections were subjected to antigen retrieval and quenching of endogenous peroxidase activity using 3% (v/v) H_2_O_2_ for 20 minutes. Immune complexes were visualized using suitable peroxidase-coupled secondary antibodies, according to the manufacturer's protocol (Histostain Plus Kit; Zymed/Invitrogen). FBPase staining was made to discriminate the proximal tubule, and double staining with *β*-catenin was performed. Additionally, to exclude false-positive results for *β*-catenin nuclear translocation, we used histological slides of *β*-catenin from the next section to perform single staining with Vulcan fast red. The whole area of the biopsy samples was examined to detect cells with nuclear localization of *β*-catenin.

### 2.10. Collagen Assay

Protein extracts from rat kidneys were used. Total collagen content was determined using the Sircol Collagen Assay kit (Biocolor, Belfast, Northern Ireland) following the enhanced protocol. Samples and collagen standards were then read at 540 nm. Collagen concentrations were calculated using a standard curve generated by using acid-soluble type 1 collagen.

## 3. Results

### 3.1. Renal Function Analysis

All animals that undergo renal mass reduction (RMR) showed clear loss of renal function that it is increasing across the time. Diuresis, proteinuria, BUN, and creatinine were highly increased in nephrectomized animals as well as urinary creatinine and creatinine clearance was markedly reduced after renal mass reduction compared with sham animals (Table 1).

### 3.2. Assessment of Renal Fibrosis in RMR Model

Left kidney from sham (S) and 5/6 nephrectomized (Nx) rats was explanted at 12 or 18 weeks after surgery and underwent qRT-PCR analysis to assess renal fibrosis. General markers for fibroblast activation including *α*-smooth muscle actin (*α*-SMA), fibroblast-specific protein-1 (FSP1), collagen 1a1 (col1a1), and vimentin (Vim) were analyzed. In addition, mRNA expression of the profibrotic mediators transforming growth factor-*β* (TGF-*β*) and plasminogen activator inhibitor-1 (Pai1) as well as the antifibrotic bone morphogenic proteins 4 and 7 (BMP4 and BMP7) were analyzed.

**Figure 1:**
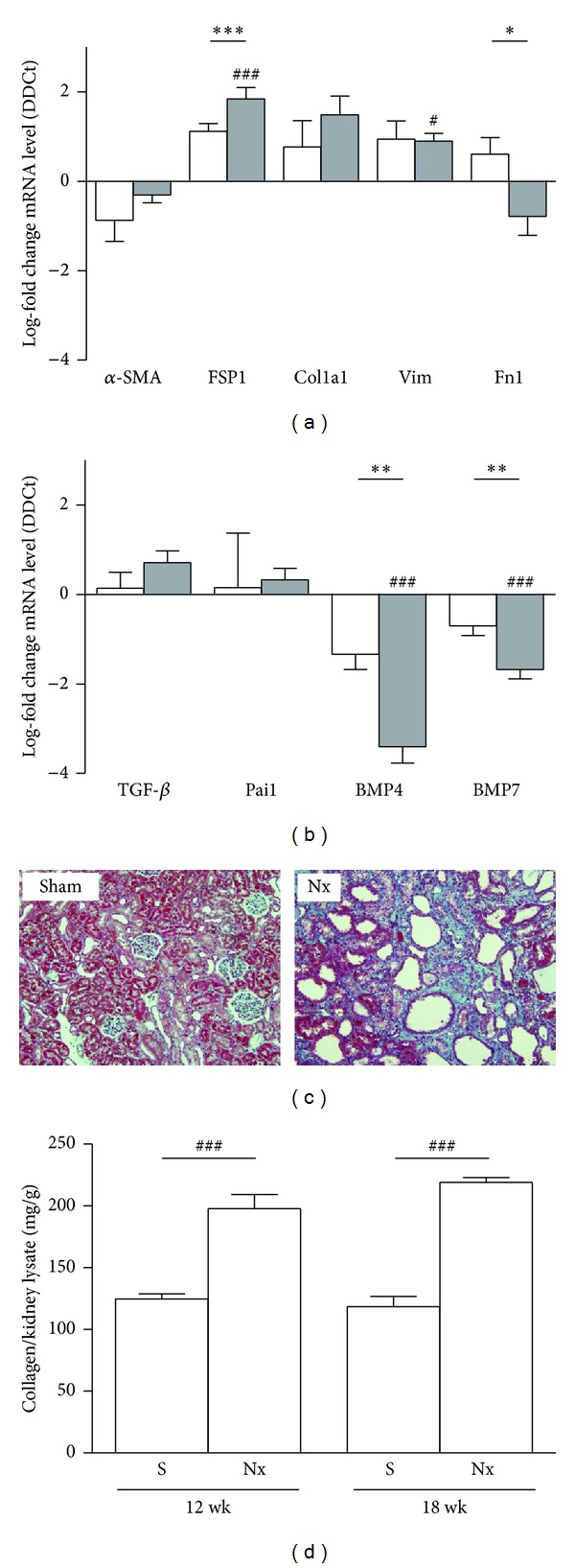
Assessment of fibrosis in rat 5/6 renal mass reduction model. (a) mRNA expression of general markers of fibroblast activation: alpha smooth muscle actin (*α*-SMA), fibroblast-specific protein-1 (FSP1), collagen 1a1 (Col1a1), vimentin (Vim), and fibronectin 1 (Fn1). (b) Profibrogenic mediators transforming growth factor-*β* (TGF-*β*), plasminogen activator inhibitor-1 (Pai1), and the antifibrotic bone morphogenetic proteins 4 and 7 (BMP4 and BMP7), and in rat kidneys of sham animals and 5/6 mass reduction animals by quantitative real-time PCR (qRT-PCR). Results are derived from 8-9 animals per group after 12 weeks (white columns) and 18 weeks (grey columns) after the surgery. Columns show median of log-fold change for each gene. ^#^ *P* > 0.05 and ^###^ *P* < 0.001, two-tailed Student's *t*-test for unpaired observations between S and Nx groups. **P > 0.01 and ***P < 0.001, two-tailed Student's *t*-test for unpaired observations between 12 and 18 weeks. (c) Masson's trichrome of representative kidneys from sham or nephrectomized (Nx) rats. Representative pictures with focus on the cortex area are given. Green: collagen deposition. (d) Total collagen content was determined using the Sircol Collagen Assay. Columns are showing show median of mg collagen/g kidney lysate. ^###^ *P* < 0.001, two-tailed Student's *t*-test for unpaired observations between S and Nx groups.

First, we analyzed the fibroblast activation and we found a progressive induction of FSP1 across time; Col1a1 and Vim showed upregulation trend; however, only Vim became statistically significant at 18 weeks (Figure 1(a)). Otherwise, *α*-SMA and Fn1 did not show expression regulation. The antifibrotic gene analysis did not show significative changes of either TGF-*β* or Pai1, while the antifibrotic proteins BMP4 and BMP7 were downregulated (Figure 1(b)).

Next we determined kidney fibrosis through quantification of collagen deposition using Masson's trichrome stain and Sircol assay. 5/6 Nx animals showed a significant increase of total collagen content compared with sham animals at 12 and 18 weeks after the surgery (Figure 1(c)).

### 3.3. Expression of the Canonical Wnt Signaling Components in RMR Model

Wnt/*β*-catenin activity was assessed during the development of fibrotic remodeling in the rat 5/6 renal mass reduction model.

**Figure 2:**
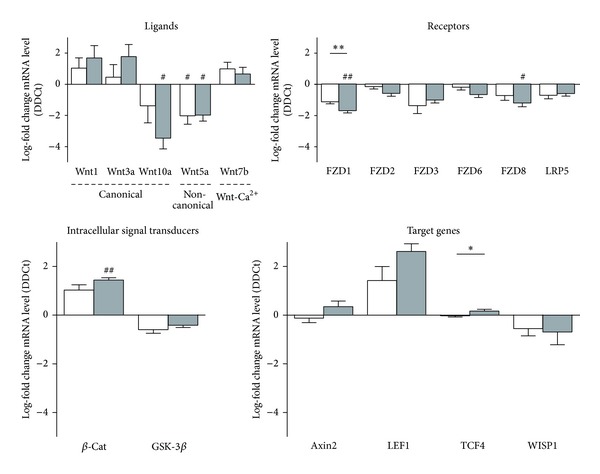
Expression of the canonical Wnt signaling components in rat 5/6 renal mass reduction model. mRNA levels of the Wnt ligands Wnt1, Wnt3a, and Wnt10a (canonical), Wnt5a (noncanonical), and Wnt7b (Ca^2+^ pathway); the Wnt receptors FZD1, 2, 3, 6, and 8 and LRP5; intracellular signal transducers *β*-catenin (*β*-Cat) and glucose sintase kinase3-*β* (GSK-3*β*); and the target genes axin2, lymphoid enhancer-binding factor 1 (LEF1), TCF4, and WISP1 were assessed in rat kidneys of sham animals (S) and 5/6 mass reduction animals (Nx) by quantitative real-time PCR (qRT-PCR). Results are derived from 8-9 animals per group after 12 weeks (white columns) and 18 weeks (grey columns) after the surgery. Columns show median of log-fold change for each gene. ^#^ *P* > 0.05 and ^###^ *P* < 0.001, two-tailed Student's *t*-test for unpaired observations between S and Nx groups. **P > 0.01 and ***P < 0.001, two-tailed Student's *t*-test for unpaired observations between 12 and 18 weeks.

At mRNA level Wnt expression of individual Wnt ligands, receptors, intracellular signal transducers, and target genes was assessed by qRT-PCR. Global analysis showed a tendency to upregulation of the pathway, but only the intracellular signal transducer *β*-catenin was significantly upregulated, whereas Wnt ligands Wnt10a and Wnt5a and receptors FZD1 and FZD8 were significantly downregulated; all other Wnt components analyzed showed tendencies (Figure 2).

**Figure 3:**
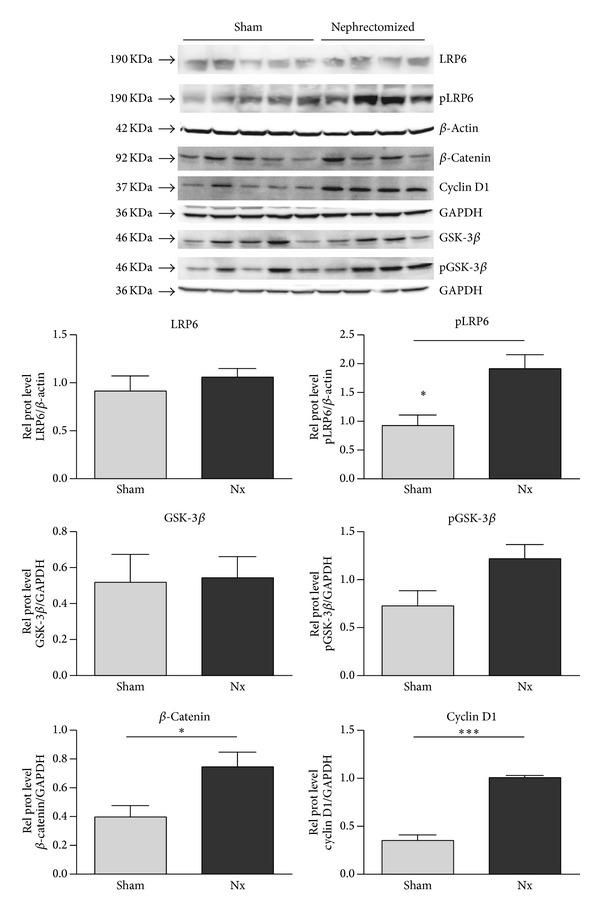
Activity of the canonical Wnt signal pathway in kidney homogenates of sham and nephrectomized rats. The expression of active Wnt components in kidney homogenates of sham and nephrectomized rats 18 weeks after the surgery was analyzed by immunoblotting of LRP6, phospho-LRP6, total *β*-catenin, cyclin D1, GSK-3*β*, and phospho-GSK-3*β*. Blotting of GDPH and *β*-actin served as loading controls. Results are derived from 4-5 animals per group.

**Figure 4:**
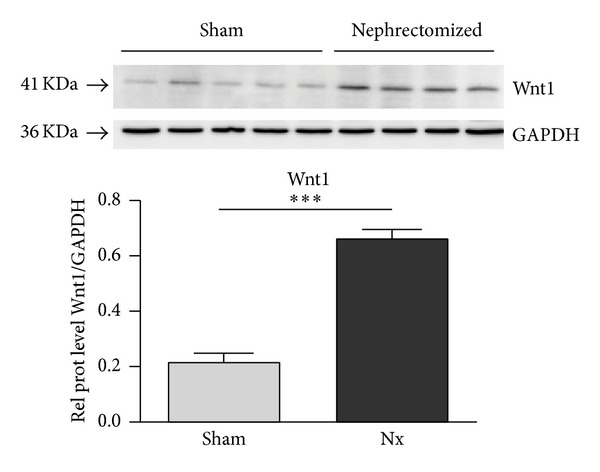
Expression of Wnt1 in kidney homogenates of sham and nephrectomized rats. The expression of Wnt1 in kidney homogenates of sham and nephrectomized rats 18 weeks after the surgery was analyzed by immunoblotting. Blotting of GDPH served as loading control. Results are derived from 4-5 animals per group.

To assess Wnt/*β*-catenin signal activity, Western blot analysis was performed to LRP6, GSK-3*β*, *β*-catenin, cyclinD1, and Wnt1 for the 18 weeks' animal groups. Phosphorylation of both LRP6 and GSK-3*β* was increased (Figure 3), indicating activation of Wnt/*β*-catenin signaling. This finding correlated with increased expression of total *β*-catenin and of the Wnt target gene, cyclin D1, both of which were upregulated in Nx rat samples as compared with kidney tissue samples from sham animals. The Wnt ligand Wnt1 at mRNA level showed a tendency to upregulation (log-fold of 2.39 ± 0.87, not statistically significant), while the Wnt1 protein level detected by Western blotting was significantly increased (Figure 4).

**Figure 5:**
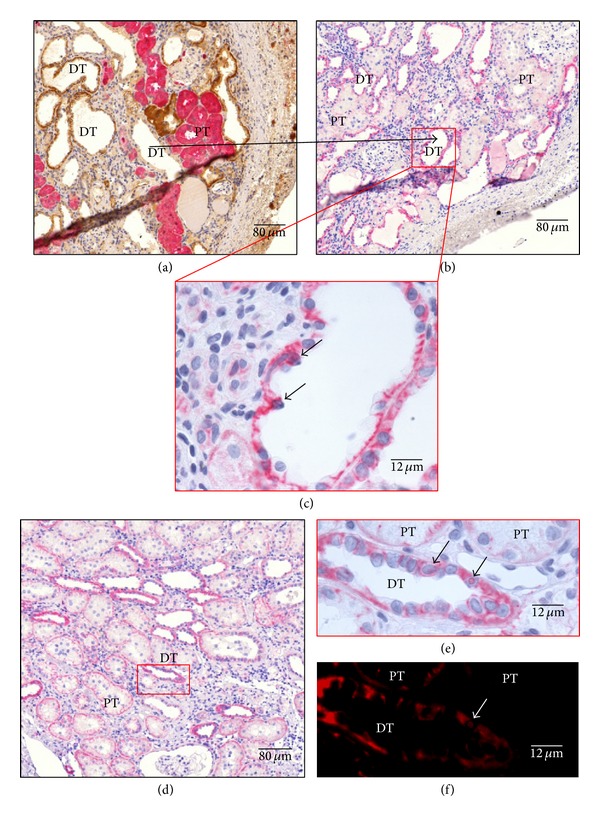
Expression and localization of *β*-catenin of representative kidneys in Nx rats. (a) Costain against FBPase in pink (Vulcan fast red), specific marker of proximal tubule (PT), and ATPase in brown (DAB), specific marker of distal tubule (DT), are made in serial cuts to discriminate between tubules; in (b) and (d) *β*-catenin stain was made in rat kidneys in pink (Vulcan fast red) and nuclei in blue (hematoxylin). Higher magnifications of tubular are shown in (c) and (e); (d) showing distal tubules with the translocated nuclei (arrows); (e) showing magnification of two proximal tubules with apical localization of *β*-catenin and one distal tubule with membrane localization, and short arrows indicate *β*-catenin translocated nucleus. As the *β*-catenin stain was made with Vulcan fast red, when slices were observed with a Texas Red filter clear image of translocated nuclei was obtained and false positives could be discarded; short arrow shows the confirmed 29 *β*-catenin translocated nucleus (f). Representative pictures with focus on the cortex area are given. Quantification of nuclei translocated *β*-catenin was done, related to proximal and distal epithelium surface. Stains are representative of using at least six different animals per group (magnification as indicated).

To further confirm pathway activation in the animal model, the *β*-catenin distribution pattern was analyzed in rat kidney. After discrimination of proximal and distal tubules (Figure 5(a)), *β*-catenin was localized to both the proximal and distal tubules (Figures 5(b)–5(d)). Notably, positive staining was limited to cell-cell junctions and the basal layer in the proximal tubule (Figure 5(e)), while the distal tubule exhibited cytoplasmic (but not nuclear) localization in sham animals. Importantly, nuclear localization of *β*-catenin was found in epithelial cells mainly on the distal tubule in Nx animals (Figures 5(c), 5(e), and 5(f)).

## 4. Discussion

Renal fibrosis is one of the most common forms in chronic kidney diseases (CKD). The goal of this study was to characterize and validate the utility of the remnant kidney model with 5/6 nephrectomy (5/6 Nx) for the study of the Wnt pathway. Histological and molecular examination of the model demonstrated the damage and the Wnt pathway activation at 12 and 18 weeks after the surgery where we can mimic CKD. This confirms to us that 5/6 Nx is an easy, reliable, and good tool where Wnt pathway is activated.

Different models of renal mass reduction have been used to study the mechanisms of the progression of CKD in humans. In 5/6 Nx, the kidney's adaptive response to this surgical reduction in nephron number appears to be close enough to the pathophysiologic characteristics of human progressive nephropathies. 5/6 Nx is a well-described low nephron number model of chronic progressive renal disease with renal function impairment, proteinuria, glomerular sclerosis, and interstitial fibrosis [15, 16]. Our group previously described histologically the model, which is characterized for arteriolar hyalinosis and glomerulosclerosis. Additionally we described glomerular hypertrophy as well as severe tubular atrophy, interstitial fibrosis, interstitial inflammation, and glomerulosclerosis [16]. Fibrosis can result from an excessive synthesis of interstitial collagens such as collagens types I and III confirmed by trichrome stain and Sircol analysis in the present study. General markers for fibroblast activation, profibrotic mediators, and antifibrotic molecules were analyzed. The differentially regulated markers of fibrosis are summarized in Table 2. Upregulation of FSP1, Col1a1, and Vim has been implicated in renal fibrosis and the downregulation of the antifibrotic BMP4 and BMP7 confirmed the fibrosis establishment, while central role of TGF-*β* in human and experimental models for renal fibrosis has been well described [3, 4, 17, 18].

It is described that stimulation of canonical Wnt pathway led to *β*-catenin accumulation, nuclear translocation, and interaction with TCF/LEF complex resulting in target gene regulation. We found upregulation at mRNA level of *β*-catenin and LEF1. The Wnt/*β*-catenin canonical pathway plays an essential role during development, driving to branching nephrogenesis in fetal kidney. In the adult, Wnt signaling plays role in the control of tissue homeostasis. Wnt pathway promotes cell proliferation, tissue expansion, cell fate determination, and terminal differentiation. mRNA analysis of Wnt ligands showed no significant upregulation of the canonical Wnt ligands Wnt1 and Wnt3a and downregulation of Wnt10b. Königshoff et al. described upregulation of Wnt1 on day 7 after obstructive injury in mice followed by a decline in mRNA levels [10]. To further confirm pathway activation at mRNA level some target genes were analyzed, and we showed they are upregulated. Table 2 shows summary of regulated genes.

When the expression distribution of *β*-catenin was analyzed in the rat renal tissue, *β*-catenin was expressed in proximal and distal tubules, but the proximal tubules showed basal localization and rarely citoplasmatic distribution, while the distal tubules showed strong cytoplasmic signal. Some nuclei of both tubules showed nuclear translocation but less frequently than in human samples. To confirm Wnt signaling activation in fibrotic rat kidneys, the key components of canonical Wnt signaling were analyzed at the protein level. Our data confirm Wnt activation, as demonstrated by an increase in the total content of *β*-catenin, LRP6, and GSK-3*β* phosphorylation, the target gene cyclin D1, and the Wnt1 ligand, indicating that Wnt1 plays a role in IFTA progression in the kidney, as previously described in heart and lung animal models. Duan et al. reported that Wnt1 was upregulated eightfold in the heart within 48 h after acute ischemic cardiac injury. These authors describe Wnt1 fibroblast proliferation and profibrotic gene induction [19]. As in the kidney, in the heart, there is low expression of most Wnt ligands, and Wnt1 enhances the profibrotic function of cardiac fibroblasts, inducing their proliferation and expression of profibrotic genes.

In our model we observed an activation of the Wnt/*β*-catenin pathway but not as strong as we expected. The 5/6 Nx model with the sacrifice at 12 and 18 weeks is a really advanced chronic damage. von Toerne et al. described CAD model and they analyzed the Wnt pathway activation across CAD development but until 8 weeks after the transplantation, and they observed upregulation across the time [2]; however this model is useful only in the transplant context. There are different types of CKD that mimic different types of CKD, glomerulosclerosis models (5/6 Nx), or interstitial fibrosis models (UUO, Cyclosporine A nephropathy, etc.). The advantages of 5/6 Nx model include robust functional readouts (such as proteinuria, GFR, and secondary hypertension), reliable induction in rodents, and comparison to the kidney removed at induction of injury. This model was useful for the elucidation of mechanisms that translate to CKD secondary to nephron loss in humans and glomerular disease [20]. He et al. described upregulation of the pathway in a UUO model but to 2 weeks after the surgery [12]. For studying tubulointerstitial fibrosis, UUO is the most widely used model. The intact nonobstructed kidney serves as an excellent control. However, this model lacks functional readouts in that serum creatinine is normal, there is no proteinuria, and 15 days could not be considered as chronic damage.

To our knowledge there are no reports about the Wnt regulation in a long term CKD. Our results demonstrate that pathway is active, but this is not a dramatic upregulation. That could be attributed to the functional tissue loss, because of the tubular and glomerular atrophy, indicating that time points analyzed could be too late for pathway modulation. Shorter time points will be necessary to set Wnt activation, in order to modulate its activation and couls study their effect on CKD activation.

## Figures and Tables

**Table 1:** Biochemical parameters for renal function evaluation.

		Diuresis	Proteinuria	Urinary creatinine	Creatinine	BUN	Creatinine
		(mL)	(mg/24 h)	(mg/24 h)	(mg/dL)	(mg/dL)	clearance
12 weeks	Sham	18.13 ± 8.25	19.88 ± 6.33	18.75 ± 2.25	0.57 ± 0.06	16.88 ± 1.25	2.32 ± 0.37
	5/6 Nx	34.88 ± 11.13	150.75 ± 65.97	12.75 ± 2.43	1.29 ± 0.31	66.63 ± 18.62	0.73 ± 0.24

18 weeks	Sham	15.37 ± 4.00	13.37 ± 4.21	19.5 ± 2.00	0.6 ± 0.06	18.38 ± 2.50	2.21 ± 0.25
	5/6 Nx	27.85 ± 6.96	143 ± 37.40	10.71 ± 3.95	1.63 ± 0.44	92.28 ± 31.77	0.53 ± 0.36

**Table 2:** Summary of differentially regulated genes.

Gene symbol	12 weeks	18 weeks	12 weeks versus 18 weeks
Fibrosis related genes			
BMP4	↓n.s.	↓	↓
BMP7	↑n.s.	*↔*	*↔*
Col1a1	↑n.s.	↑n.s.	↑n.s.
FSP1	↑n.s.	↑	↑
Vim	↑n.s.	↑	*↔*
Wnt pathway ligands			
Wnt1	↑n.s.	↑n.s.	↑n.s.
Wnt3a	↑n.s.	↑n.s.	↑n.s.
Wnt5a	↓n.s.	↓n.s.	*↔*
Wnt7b	↑n.s.	↑n.s.	*↔*
Wnt10a	↓n.s.	↓n.s.	*↔*
Receptors			
FZD1	↓n.s.	↓	↓
FZD3	↓n.s.	↓n.s.	*↔*
FZD6	*↔*	↓n.s.	*↔*
FZD8	↓n.s.	↓	*↔*
LRP5	↓n.s.	↓n.s.	*↔*
Intracellular signal transducers			
*β*-Cat	↑n.s.	↑	↑
GSK-3*β*	↓n.s.	↓n.s.	↑n.s.
Target			
Axin2	↑n.s.	*↔*	*↔*
LEF1	↑n.s.	↑	*↔*
WISP1	*↔*	*↔*	↑n.s.

↑: statistically significant upregulation.

↓: statistically significant downregulation.

↑n.s.: no statistically significant upregulation.

↓n.s.: no statistically significant downregulation.
